# Major intrinsic protein (*MIP*) polymorphism is associated with age-related cataract in Chinese

**Published:** 2011-08-25

**Authors:** Zhou Zhou, Binbin Wang, Yongfeng Luo, Guangkai Zhou, Shanshan Hu, Han Zhang, Xu Ma, Yanhua Qi

**Affiliations:** 1 Department of Ophthalmology, the Second Affiliated Hospital of Harbin Medical University, Harbin, China; 2 Department of Genetics, National Research Institute for Family Planning, Beijing, China; 3 2007 Clinical Medicine, Harbin Medical University, Harbin, China

## Abstract

**Purpose:**

Age-related cataract (ARC) is a complex multi-factorial disorder involving several genetic and environmental factors. The major intrinsic protein of lens fiber gene (*MIP*) encodes the most abundant junctional membrane protein in the mature lens and plays a critical role in maintainace of lens normal structure and internal circulation. To determine the relationship between single nucleotide polymorphisms (SNPs) in *MIP* and the susceptibility to ARC in a Chinese population, we conducted this case-control study.

**Methods:**

A total of 164 unrelated ARC patients and 132 normal controls were involved in the study. All participants completed full physical and ophthalmic examinations and provided a blood sample for DNA extraction. Seven SNPs (rs2269348, rs61759527, c.-4T>C, rs77163805, rs74641138, rs35033450, and rs36032520) in *MIP* were amplified by polymerase chain reaction (PCR) and then sequenced. Statistical analysis was performed using SNPstats.

**Results:**

Polymorphisms rs61759527, rs77163805, rs35033450, and rs36032520 were not detected in all 296 subjects. There were no statistical differences in genotype or allele frequency of rs2269348 and rs74641138 between ARC cases and controls. But in c.-4C>T, cataract patients had a higher TC genotype and C allele frequencies (p=0.0018 and p=0.017, respectively) compared to healthy controls. The haplotype CCG of rs2269348, c.-4T>C and rs74641138 also exhibited a signiﬁcantly higher distribution in cases than controls (OR=8.83, p=0.0024).

**Conclusions:**

Our findings indicate that the genotype TC in polymorphism c.-4T>C and haplotype CCG of rs2269348, c.-4T>C, and rs74641138 in *MIP* may attach an additional genetic risk factor for ARC in Chinese. This is the first association study about SNPs in *MIP* and susceptibility to ARC in Chinese population.

## Introduction

Age-related cataract (ARC) is the leading cause of low vision and blindness all over the world [1]. As the world’s population ages, cataract-induced visual dysfunction and blindness is on the increase [2]. The development of ARC is complex and multifactorial; the risk factors for cataract including being of the female sex, having lower socioeconomic status, having diabetes mellitus, smoking, and lower body mass index [3-5]. Recently, the contribution of genetic factors in the pathogenesis of ARC was confirmed by some twin studies. In 2000 and 2001, Hammond et al. recognized the heritability for age-related cortical cataract was 53%–58% [6] and approximately 48% for nuclear cataract [7]. Furthermore, several genes were proven to link to ARC, including EPH receptor A2 (*EPHA2*, chromosome 1p) [8], gap junction protein α8 (*GJA8*, chromosome 1q) [9], galactose-1-phosphate uridylyltransferase (*GALT*, chromosome 9p) [10],solute carrier family 16, member 12 (*SLC16A12*, chromosome 10q) [11], heat shock transcription factor 4 (*HSF4*, chromosome 16q) [12], galactokinase 1 (*GALK1*, chromosome 17q) [13], ferritin light polypeptide (*FTL*, chromosome 19q) [14], xeroderma pigmentosum complementation group (*XPD*, chromosome 19q), X-ray complementing group1 (*XRCC1*, chromosome 19q) [15], crystallin αA (*CRYAA*, chromosome 21q) [16], crystalline βB2 (*CRYBB2*, chromosome 22q) [17], and the glutathione-S-transferases family (*GSTM1*, chromosome 1p, *GSTP1*, chromosome 11q, and *GSTT1*, chromosome 22q) [18-20].

The lens is a major component of the dynamic vision system. It develops and maintains a tissue structure predicated on the formation and precise organization of specialized cells, the lens ﬁbers. As an avascular tissue, the maintenance of homeostasis and normal structure in lens fiber cells is highly dependent on the channels and adhesion molecules on the cells membrane [21]. The major intrinsic protein of lens fiber gene (*MIP*), which is also called aquaporin0 (*AQP0*), encods the most abundant junctional membrane protein in the mature lens. MIP functions not only as a lens-specific water channel but also a cell-to-cell adhesion molecule [22]. Mutations in *MIP* have been linked to mouse and human congenital cataracts, but there was no report about the association between the single nucleotide polymorphisms (SNPs) in *MIP* and ARC until now. In this study, we tested seven SNPs in *MIP* for association with ARC in a Chinese case-control cohort. We substantiated that cataract patients had a higher TC genotype and C allele frequencies in c.-4T>C (p=0.0018 and p=0.017, respectively) compared to healthy controls. The haplotype CCG of rs2269348, c.-4T>C and rs74641138 exhibited a signiﬁcantly higher distribution (OR=8.83, p=0.0024) in cases than controls as well. Our results suggested there may be an association between the SNP c.-4T>C in *MIP* and the susceptibility of ARC in the Chinese population.

## Methods

### Patients and controls

Patients with age-related cataract and normal controls were collected from the northeast of China during our clinical work. Cataract diagnosis was determined according to lens opacities classification system III (LOCSIII) [23]. Patients with secondary cataracts due to trauma, toxins, inflammation, and degenerative ocular diseases were excluded from the study. In addition, patients with diabetes, hypertension, high myopia, glaucoma, any syndrome, and those who were a smoker, an alcoholic, exposed to UVB radiation, or under medication like steroids were also not considered. The control subjects from the same ethnic background were selected during routine medical fitness examination which included ophthalmic examination. From all the patients included in the study, information pertaining to sex, age, age at onset, and family history were collected using a specified form.

Informed consent was obtained from all subjects after explaining the nature and possible consequences of the study. All experiments were approved by the Institutional Review Board of Harbin Medical University (Harbin, China) and conducted according to the principles in the Declaration of Helsinki. To date, all of the patients have had cataract surgery.

### SNP selection

We used the SNP database at NCBI and selected SNPs in the 5′UTR, exon 1, and exon 2 of *MIP* for this study. Six SNPs were chosen for genotyping, including rs2269348, rs61759527 (c.-10C>A), rs77163805 (c.199G>A, p.67V>I), rs74641138 (c.319G>A, p. 107V>I), rs35033450 (c.378C>T) and rs36032520 (c.516C>T). In the process of this study, we found a polymorphism c.-4C>T which had no record in the NCBI SNP database but was in 1000 Genomes and we added it into our project.

### DNA extraction and genotyping

Five milliliter of venous blood samples were collected in EDTA vaccutainers from ARC patients and control subjects. Genome DNA was extracted from peripheral blood leukocytes with QIAamp DNA Blood Mini Kits (QIAGEN Science, Germantown, MD). The first exon in *MIP* and its flanking sequences were amplified by polymerase chain reaction (PCR). The primers used in the reaction were 5′-TCT CGG CTC ATC TCC CAG TT-3′ (forward), 5′-GGC AAT AGA GAG ACA GGA CAC-3′ (reverse) and 5′-TGA AGG AGC ACT GTT AGG AGA TG-3′ (forward), 5′- AGA GGG ATA GGG CAG AGT TGA TT −3′ (reverse). The primers were designed with Primer3 according to the reference sequences in the NCBI. We sequenced the PCR products using an ABI3730 Automated Sequencer (PE Biosystems, Foster City, CA), and analyzed the sequencing results using Lasergene SeqMan (DNASTAR, Madison, WI).

### Statistical analysis

Allele and genotype frequencies of *MIP* rs2269348, rs61759527, c.-4T>C, rs77163805, rs74641138, rs35033450, and rs36032520 were compared between patients and controls. We evaluated the frequency of genotypes and alleles in this study using the χ^2^ test and logistic regression. Paired SNP linkage disequilibrium analysis and haplotype analysis were done as well. The risk for patients was estimated using the odds ratio (OR) and 95% confidence interval (CI). Statistical analysis was performed using SNPstats [24]. A p<0.05 was considered statistically signiﬁcant.

## Results

### Characteristics of patients and controls

A total of 296 individuals were included in this study (Table 1). Of these, 164 unrelated individuals were ARC patients with a mean onset age of 66.9±7.3 years. In addition, 132 unaffected individuals with a mean age of 54.2±9.0 years were also recruited. There were significantly more females than males in ARC patients (106:58), and a higher percentage of females overall compared with males (194:102). The patients and controls were matched on gender (χ^2^ test, p=0.70). The difference of age was adjusted by logistic regression.

**Table 1:** Baseline characteristics of the study subjects.

**Group**	**Total**	**Male(%)**	**Female(%)**	**Mean age**
Age-related cataract case	164	58 (35.3)	106 (64.7)	66.9±7.3
Control	132	44 (33.3)	88 (67.7)	54.2±9.0
			χ^2^ test, p=0.70	*t*-test, p<0.05

### Genotype and allele frequencies

The distribution of all the SNPs were consistent with the Hardy–Weinberg equilibrium (HWE) in both case and control groups. Polymorphisms rs61759527, rs77163805, rs35033450, and rs36032520 were not detected in all 296 subjects.

Table 2 showed the distribution of the genotype and allele frequencies of rs2269348, c.-4T>C, and rs74641138 in the study population. Compared with controls, a significant higher distribution of ARC cases was observed in either the TC genotype frequency [OR (95%CI)=6.18 (1.84–20.78), p=0.0018] or C allele frequency (χ^2^=5.731, p=0.017) in c.-4T>C. There were no statistical differences in the rs2269348 and rs74641138 genotype and allele frequencies between ARC cases and controls.

**Table 2:** Distribution of the genotype and allele frequencies of rs2269348, c.-4T>C, and rs74641138 in the case and control group (adjusted by age).

**SNP**		**Genotype N (%)**			**Allele N (%)**	
**rs number**	**Variation**	**Group**	**TT**	**TC**	**CC**	**T**	**C**
rs2269348		Case	73 (44.5%)	73 (44.5%)	18 (11%)	219 (67%)	109 (33%)
		Control	72 (55%)	51 (39%)	9 (7%)	195 (74%)	69 (26%)
			p=0.078 OR (95%CI)=3.51 (1.07–11.50)			χ^2^=3.502,p=0.061	
			TT	TC	CC	T	C
-	c. −4T>C	Case	144 (87.8%)	20 (12.2%)	0	307 (94%)	21 (6%)
		Control	126 (95.5%)	6 (4.5%)	0	258 (98%)	6 (2%)
			p=0.0018 OR (95%CI)=6.18 (1.84–20.78)			χ^2^=5.731, p=0.017	
			GG	GA	AA	G	A
rs74641138	c. 319G>A (p.V107I)	Case	155 (94.5%)	9 (5.5%)	0	319 (97%)	9 (3%)
		control	125 (94.7%)	7 (5.3%)	0	257 (97%)	7 (3%)
			p=0.77 OR (95%CI)=1.24 (0.29–5.40)			χ^2^=0.005, p=0.943	

### Paired SNP linkage disequilibrium analysis and haplotype analysis

**Figure 1:**
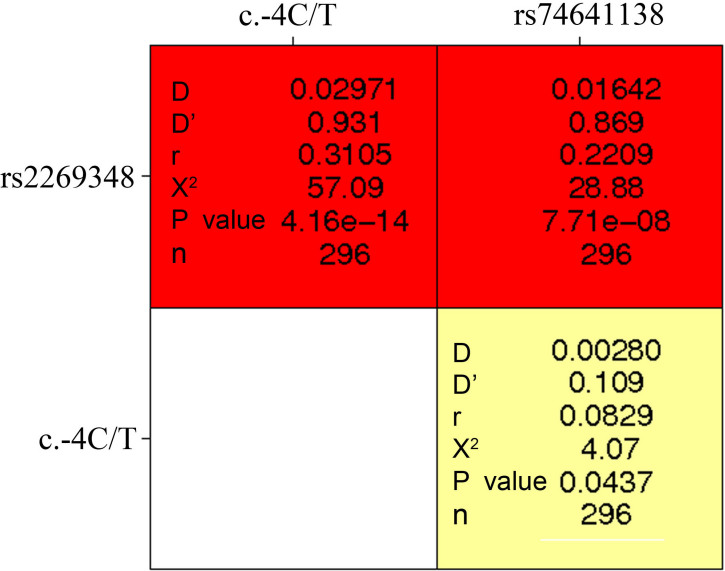
Paired SNP linkage disequilibrium analysis. Paired SNP linkage disequilibrium analysis revealed that rs2269348 was in linkage disequilibrium with c.-4T>C and rs74641138 (D’>0.75).

Paired SNP linkage disequilibrium analysis revealed that rs2269348 was in linkage disequilibrium with c.-4T>C and rs74641138 (D’>0.75; Figure 1). Haplotype case–control analysis denoted that the haplotype CCG with the C allele of rs2269348, the C allele of c.-4T>C, and the G allele of rs74641138 exhibited a signiﬁcantly higher distribution in cases than controls (OR=8.83,p=0.0024; Table 3).

**Table 3:** The haplotype association analysis (adjusted by age).

rs2269438	**c.-4T>c**	rs74641138	**Case frequency**	**Control frequency**	**OR (95% CI)**	**p value**
T	T	G	0.6677	0.7281	1.00	—
C	T	G	0.2474	0.2226	1.34 (0.78–2.31)	0.29
C	C	G	0.0575	0.0173	8.83 (2.19–35.64)	0.0024
C	T	A	0.0209	0.0215	1.63 (0.27–9.70)	0.59

## Discussion

In this study, we investigated the association of *MIP* polymorphisms with ARC in the Chinese Han population and found a statistically significant difference between cases and controls. There was an association between the gene polymorphisms of *MIP* and the susceptibility to ARC.

The adult-onset and progressive pattern of inherited cataracts suggested the majority of age-related cataract-associated genes are hereditary cataract candidate genes [25]. So far, several congenital cataract-causing genes are reported to contribute to ARC. Variants in *EPHA2* were shown to associate with age-related cortical cataract in both human and mice [8]. Targeted knockout of the mouse *Crybb2* can induce age-related cataract in mice [17]. A mutation (F71L) causing defective chaperone-like function in αA-crystallin was reported to be associated with ARC [16]. Shi et al. [12] identiﬁed ﬁve new *HSF4* mutations in 150 ARC patients, ﬁnding that these mutations might account for a small fraction of age-related cataracts. Recently, polymorphisms in *GJA8* were found to associate with ARC in Chinese people [9]. In *SLC16A12*, a heterozygous sequence alteration in the 5′UTR (c.-17A>G), which was found in an ARC patient, is in cis with the minor G allele in SNP rs3740030 (c.-42T>G) [11].

*MIP* (OMIM 154050) was initially a candidate gene for hereditary cataract. Its product, which is also called AQP0, is expressed in lens fiber cell membranes. It is critical for lens transparency and homeostasis as *MIP* mutations in human and mice have resulted in dominant lens cataract [26,27]. The function of MIP, both as a water channel and an adhesive molecule in the lens fiber, contribute to the narrow intercellular space of the lens fibers that is required for lens transparency and accommodation [8]. Moreover, it is involved in the maintenance of fiber-fiber interactions that regulated the morphology and arrangment of lens fiber cells [28] and could interact with many other lens components including crystallins, lipids, and cytoskeletal proteins [29-31]. With age, significant modifications of AQP0 have already been characterized in human lenses [32]. A continuous age-dependent process of AQP0 truncation was observed and it may contribute to the formation of the lens barrier [33]. As suggested above, AQP0 should play an important role in the process of age-related cataract.

In this study, we found the association of SNP c.-4T>C and haplotype CCG of rs2269348, c.-4T>C and rs74641138 in *MIP* with ARC, which is presumed to be responsible for the molecular changes leading to cataract formation in aged people. The TC heterozygotes is obviously higher in patients with an odds ratio of 6.18 (95%CI=1.84–20.78, p=0.0018). The haplotype CCG of rs2269348, c.-4T>C, and rs74641138 showed a significant higher frequency in patients than controls with an OR of 8.83 (95%CI=2.19–35.64, p=0.0024). These indicated that the TC genotype and CCG haplotype possibly increased the diease risk. The functional consequences of the c.-4T>C polymorphism is unknown yet. Since it lays in the 5′UTR, 4 bases upstream from the start codon, it may influence the efficiency of protein translation. Previous researches showed that disturbance of the regulation of the translational machinery leads to perturbed cellular metabolism and may tilt the physiologic balance from healthy to diseased states [34]. Linkage disequilibrium analysis revealed that rs2269348, c.-4T>C, and rs74641138 are in linkage disequilibrium. More experiments are needed to explore whether the C allele work alone or with the other alleles in haplotype CCG. We need further studies to find out the precise mechanisms by which the polymorphisms influence the natural history of age-related cataract development.

This is the first association study about *MIP* gene polymorphisms and age-related cataract in Chinese people. However, the etiology and pathogenesis of ARC is complex and has multiple factors. More research should be done to investigate the relationship between *MIP* and ARC.
